# Combined Effects of Low-Density Polyethylene (LDPE), Zn(II), Cu(II), and Metolachlor on *Trichoderma harzianum* Growth, Oxidative Stress Induction, and Herbicide Degradation

**DOI:** 10.3390/molecules31061038

**Published:** 2026-03-20

**Authors:** Anastasiia Kubera, Przemysław Bernat, Sylwia Różalska, Alicja Okrasińska, Mirosława Słaba

**Affiliations:** 1 Doctoral School of Exact and Natural Sciences, Faculty of Biology and Environmental Protection, University of Lodz, Banacha Street 12/16, 90-237 Lodz, Poland; anastasiia.kubera@edu.uni.lodz.pl; 2 Department of Industrial Microbiology and Biotechnology, Faculty of Biology and Environmental Protection, University of Lodz, Banacha Street 12/16, 90-237 Lodz, Poland; przemyslaw.bernat@biol.uni.lodz.pl (P.B.); sylwia.rozalska@biol.uni.lodz.pl (S.R.); 3 University of Warsaw, Biological and Chemical Research Centre, Faculty of Biology, Institute of Evolutionary Biology, Zwirki i Wigury 101, 02-089 Warsaw, Poland; a.okrasinska@uw.edu.pl

**Keywords:** microplastics, low-density polyethylene, metolachlor, *Trichoderma harzianum*, heavy metals, Zn(II), Cu(II), herbicide degradation, reactive oxygen species

## Abstract

The widespread presence of microplastics (MPs), heavy metals, and herbicide residues in agricultural soil raises concerns about their combined effects on soil microorganisms. This study examined the combined impact of Zn(II)/Cu(II), low-density polyethylene (LDPE), and metolachlor (MET) on *Trichoderma harzianum* IM 7002, a strain isolated from heavily polluted soil in central Poland. Exposure to LDPE and MET alone reduced fungal growth and induced oxidative stress, whereas Zn(II) at a concentration of 5 mM and Cu(II) at a concentration of 2.5 mM stimulated growth and enhanced MET degradation. HPLC MS/MS analysis identified transformation products, confirming active degradation even under co-exposure to LDPE and metals. Notably, simultaneous exposure to MET, LDPE, and Cu(II) (5 mM) increased antioxidant enzyme activity and decreased lipid peroxidation, suggesting a strengthened antioxidant defense and/or partial utilization of reactive oxygen species during MET biotransformation. Pollutant mixtures also caused quantitative shifts in membrane phospholipid composition and a slight increase in membrane permeability, indicating both toxic effects and adaptive membrane remodeling in response to chemical stress. Overall, *T. harzianum* IM 7002 exhibited high tolerance to complex pollutant mixtures while maintaining herbicide-degradation capacity, highlighting its potential for remediation of contaminated agricultural soils.

## 1. Introduction

Plastic pollution was long considered a problem primarily affecting marine environments [1]. However, recent research has demonstrated that it is now widespread across diverse ecosystems, including deserts, mountain peaks, oceans [2], rivers, surface lakes, food, the atmosphere, and soils (e.g., farmland) [2,3]. Cusworth et al. [4] reported that annual plastic production has reached approximately 450 million tons, of which 12.5 million tons are used in the agricultural sector. Alarmingly, around 76% of produced plastics become waste, and 79% of this fraction accumulates in landfills or the natural environment. Due to their persistence, plastics may require several decades to several centuries to decompose under natural conditions [1,2].

Environmental plastics are commonly classified into three groups according to size: macroplastics (>5 mm), microplastics (<5 mm, MPs), and nanoplastics (<1 μm or 1000 nm) [4,5]. Among these, MPs are the most frequently detected and therefore become a major focus of research [3,5]. Microplastics pose a serious threat to ecosystems because they can disrupt soil ecology and reduce agricultural productivity [6].

Microplastics enter agricultural soils through multiple pathways, including plastic mulching, crop covers, wastewater irrigation, polymer-coated agrochemicals, and atmospheric deposition [4,7]. Once deposited, they fragment into smaller particles, thereby altering the soil structure [4], as well as physicochemical properties [6], fertility, and overall functioning [8]. They also influence soil microbial communities [9]. For example, increased accumulation of plastic residues has been associated with reduced carbon and nitrogen content in soil microbial biomass. MPs can disrupt soil structure and can negatively impact soil microorganisms, limiting their growth and their ability to fix carbon and nitrogen [7].

Moreover, soil microorganisms can degrade both biodegradable and non-biodegradable plastics, although these materials are not their primary substrates [10]. Previous studies have also reported changes in fluorescein diacetate hydrolase activity in microplastic-contaminated soils [9]. Nevertheless, the effects of MPs on soil microbial communities are complex and remain insufficiently understood, highlighting the need for further investigation [6,7].

Pesticides are among the most important organic pollutants due to their widespread use in agriculture. In soils, MPs can adsorb pesticides, thereby influencing their persistence and environmental fate. Such interactions may pose risks to soil quality, particularly when pesticides are transported together with microplastics across ecosystems [11]. Although the occurrence and behavior of MPs and pesticides have been described in the literature, research on the combined effects and toxicity remains limited. Our previous studies showed that the presence of MPs in herbicide-contaminated soil accelerated herbicide degradation by *Trichoderma harzianum* [7]. Other reports indicate that small plastic particles readily accumulate pesticides in soils [12], and several studies have shown that pesticides such as acetamiprid and flubendiamide can be adsorbed on the surfaces of MPs [13]. Guo et al. [14] found that pesticide concentrations in plastic mulch film residues were approximately 20 times higher than in surrounding soils, suggesting a potential hazard from pesticide-associated plastic particles and highlighting unknown mechanisms of their impact within the soil profile that warrant closer attention.

In addition to pesticides, heavy metals represent another group of widespread environmental pollutants. They can occur naturally in soils (e.g., via organic residues and geological processes) or originate from anthropogenic sources such as agricultural activities [15]. In combination with MPs, heavy metals pose a persistent and severe environmental threat. Studies have documented that MPs can sorb heavy metals [16,17], which are subsequently transported into sediments and groundwater, exerting long-lasting effects on natural ecosystems [17]. However, the combined effects of MPs, heavy metals, and metolachlor (MET) in agricultural soils and on soil microbiota are poorly understood. Fungi of the genus *Trichoderma* play essential roles in agriculture as key biocontrol organisms and plant-growth promoters acting through nutrient mobilization, production of antifungal metabolites, and stimulation of plant defense pathways. Their beneficial activity strongly depends on soil conditions. Consequently, evaluating how combined pollutants affect *Trichoderma* is critical for understanding the stability and functionality of biocontrol systems in modern agriculture.

The aim of the present study was to evaluate the combined effects of Zn(II) or Cu(II) and low-density polyethylene (LDPE) on the growth of *T. harzianum* IM 7002 and on MET degradation. Additionally, we assessed whether LDPE and Zn(II) or Cu(II), applied individually or in combination, induce oxidative stress in fungal cells, by measuring the activities of antioxidant enzymes, including catalase (CAT) and superoxide dismutase (SOD). Changes in membrane phospholipid composition and membrane permeability were also analyzed.

## 2. Results

### 2.1. Isolation and Identification of a Metolachlor Degrading Fungal Strain

**Figure 1:**
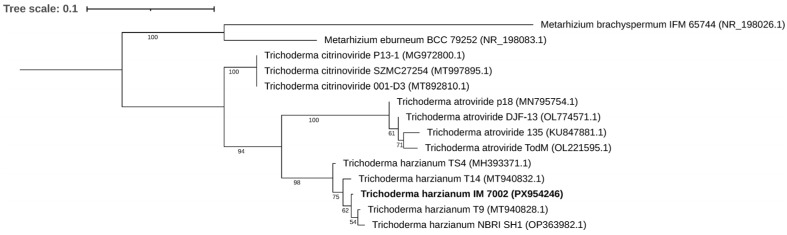
Phylogenetic relationships of 12 *Trichoderma* strains and 2 *Metarhizium* strains based on ITS sequences. The numbers at the nodes indicate the level of bootstrap support (%) based on maximum likelihood analysis of 1000 re-sample datasets.

Querying the NCBI Nucleotide database with the obtained ITS rRNA sequence of the IM 7002 strain suggested that the strain belongs to the *Trichoderma* genus, most likely *T. harzianum*, *T. atroviride*, or *T. citrinoviride*. Further analysis, i.e., reconstructed ITS rRNA phylogeny, placed the strain within *T. harzianum* species (Figure 1).

### 2.2. Effect of Metolachlor and Heavy Metals on Spore Viability and Biomass of T. harzianum

**Figure 2:**
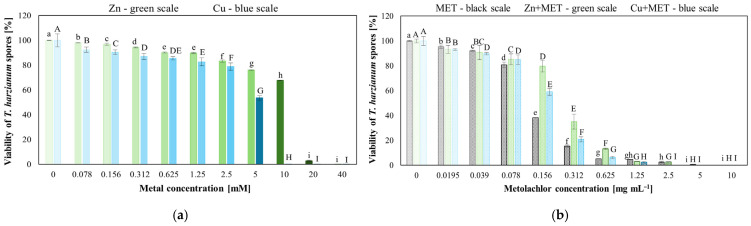
Spore viability of *Trichoderma harzianum* IM 7002 exposed to: (**a**) Zn(II) and Cu(II) (lowercase letters—significant differences among Zn(II) treatment; uppercase letters—significant differences among Cu(II) treatment); (**b**) metolachlor (MET) alone or combined with Zn(II) and Cu(II) (metal concentrations correspond to those shown in (**a**); lowercase letters—significant differences among MET treatment; uppercase letters—significant differences among Zn(II) or Cu(II) treatment). Different letters indicate statistically significant differences (*p* < 0.05, one-way ANOVA followed by Dunnett’s test).

The viability of *T. harzianum* IM 7002 spores depended on both the type and concentration of chemical stressors, including Zn(II), Cu(II), MET, and their combination. Spore viability decreased significantly with increasing concentrations of all tested substances (*p* < 0.05). Under Zn(II) exposure, viability remained above 67% up to 10 mM but dropped sharply to 3% at 20 mM. Cu(II) exhibited stronger toxicity, with spore viability declining to 53% at 5 mM, and at 10 mM, spores completely lost viability. MET reduced viability to 39% at 0.156 g L^−1^ and to 0.6% at 5 g L^−1^. Co-application of MET with higher concentration Zn(II) or Cu(II) further enhanced the toxic effect on spore viability (Figure 2).

**Figure 3:**
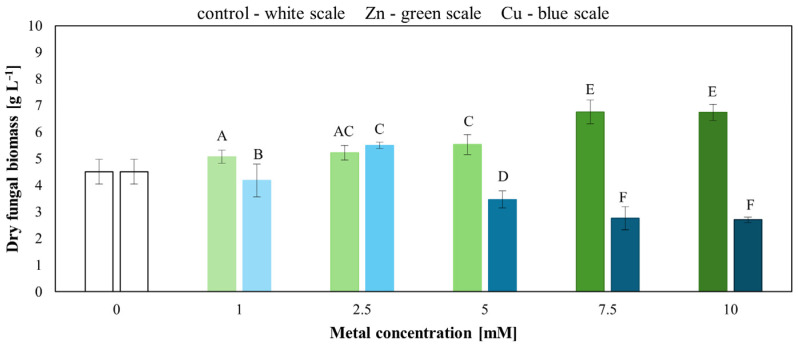
Biomass production of *T. harzianum* in liquid medium supplemented with Cu(II) or Zn(II). Letters indicate statistically significant differences (uppercase letters—significant differences depending on the Zn(II) or Cu(II) concentration). Statistical significance was determined by one-way ANOVA followed by Dunnett’s test vs. control and post hoc Tukey’s HSD test for comparisons between Zn(II)/Cu(II) (*p* < 0.05).

Increasing concentrations of Zn(II) and Cu(II) also affected the growth of *T. harzianum* IM 7002. Statistical analysis indicated that all treated samples differed significantly from the metal-free control (*p* < 0.05), except for 1 mM Cu(II), which did not differ significantly. The highest biomass yields (g L^−1^) were observed at 10 mM of Zn(II) and 2.5 mM of Cu(II), suggesting that these concentrations may stimulate the growth of *T. harzianum* IM 7002 (Figure 3). In contrast, concentrations above 10 mM Zn(II) and 5 mM Cu(II) inhibited fungal growth, confirming the toxic effect of higher metal concentrations (*p* < 0.05).

### 2.3. Degradation of Metolachlor by T. harzianum in the Presence of LDPE and Zn(II) or Cu(II)

**Figure 4:**
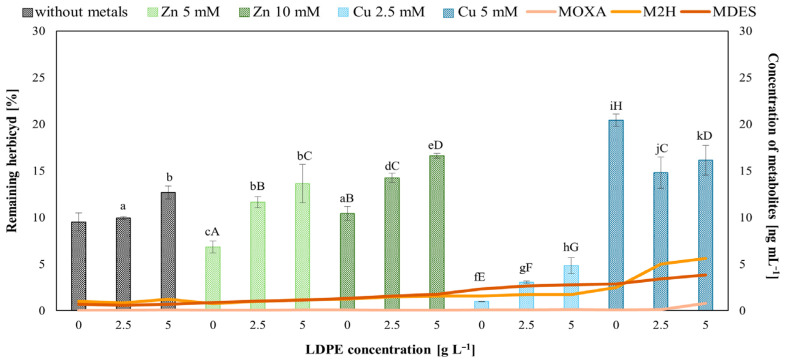
Concentrations of MET and its metabolites in liquid medium after 7 days of *T. harzianum* IM 7002 cultivation. Abiotic controls consisted of medium supplemented with MET, 50 mg L^−1^ and/or, depending on the experimental variant, low-density polyethylene (LDPE) and/or metal, incubated for 7 days under the same conditions). Different letters indicate statistically significant differences (*p* < 0.05, two-way ANOVA followed by post hoc Tukey’s HSD test). Lowercase letters—denote significant effects of LDPE; uppercase letters—denote significant effects of Zn(II) or Cu(II); MDES—metolachlor deschloro; M2H—metolachlor-2-hydroxy; MOXA—metolachlor oxanilic acid.

Previous studies have shown that *T. harzianum* can degrade MET [18]. Environmental pollutants, including LDPE and Zn(II) or Cu(II), may modulate this process. In the present study, *T. harzianum* IM 7002 reduced MET content by approximately 90% relative to the abiotic control (Figure 4).

After 7 days of incubation LDPE (5 g L^−1^) and both metals (Zn(II) or Cu(II)) significantly affected MET degradation (main effects, *p* < 0.05). Zn(II) at 5 mM enhanced degradation, while Cu(II) (5 mM) impaired it. LDPE alone reduced MET elimination. Significant interaction effects were observed between metal concentration and LDPE; the enhancing effect of Zn(II) was diminished by LDPE, whereas the inhibitory effect of higher Cu(II) concentration was further exacerbated when combined with LDPE.

The HPLC-MS/MS analysis indicated the formation of MET degradation products, including metolachlor deschloro (MDES), metolachlor-2-hydroxy (M2H), and metolachlor oxanilic acid (MOXA). These metabolites were identified based on their mass spectra (*m*/*z* 148 and 176), which were consistent with literature data and with the MET reference spectrum [19]. The highest amounts of MET metabolites were detected in *Trichoderma* cultures simultaneously exposed to 5 mM Cu(II) and LDPE. This observation indicates that the strain retains the capacity for enzymatic herbicide transformation even under 5 mM Cu(II). Statistical analysis using the post hoc Tukey’s HSD (*p* < 0.05) showed significant differences between all tested systems and the control (without metals or LDPE), except for treatment containing LDPE alone (2.5 g L^−1^) and Zn(II) alone (10 mM).

### 2.4. Mycelial Biomass and the Ergosterol Content of T. harzianum During Herbicide Degradation in the Presence of LDPE and Zn(II) or Cu(II)

**Figure 5:**
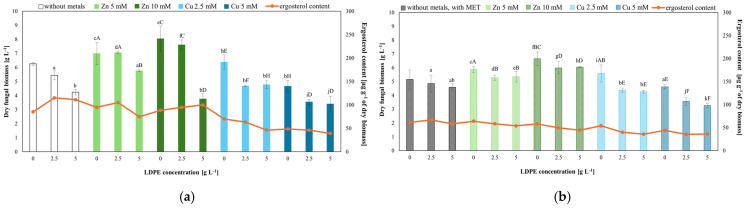
Dry biomass and the ergosterol content of *T. harzianum*: (**a**) without (solid-colored bars) and (**b**) with metolachlor (cross-hatched bars). Different letters indicate statistically significant differences (*p* < 0.05, two-way ANOVA followed by post hoc Tukey’s HSD test). Lowercase letters—statistically significant effects of LDPE; uppercase letters—statistically significant effect of Zn(II) or Cu(II) concentration.

Dry fungal biomass and the ergosterol content were determined to evaluate the physiological status of *T. harzianum* under exposure to LDPE, Zn(II), or Cu(II) and MET. LDPE alone reduced the fungal growth, and the effect was intensified when they were combined with MET, indicating the inhibition of fungal metabolism (Figure 5). Zn(II) at the tested concentrations increased dry biomass (up to 8.044 g L^−1^); however, fungal growth was limited in the presence of LDPE. Cu(II) reduced growth, with a further decline observed upon co-exposure to LDPE (Figure 5a). Notably, the combined highest concentrations of Cu(II) combined with LDPE caused the most pronounced reduction in biomass (Figure 5).

The post hoc Tukey’s HSD test (*p* < 0.05) revealed significant differences between the tested systems and the control lacking LDPE and Zn(II)/Cu(II). However, no significant differences were observed for Zn(II) (10 mM) combined with LDPE (5 g L^−1^) or for Cu(II) (2.5 mM). The same test (*p* < 0.05) also indicated significant differences between MET-amended systems and their corresponding MET-free controls (Table S1, Supplementary Materials). No significant differences were found for LDPE (2.5 g L^−1^) alone or for Zn(II) (10 mM) in combination with LDPE (2.5 and 5 g L^−1^).

Ergosterol content generally reflected the biomass trends. LDPE alone caused an increase in the amount of ergosterol, where MET reduced ergosterol content, indicating that the toxicity of Zn(II) and MET did not substantially affect ergosterol biosynthesis in the absence of MET. However, under MET exposure, ergosterol production was markedly reduced. Cu(II) caused a decrease in the ergosterol content, and this reduction was further enhanced under combined exposure to LDPE and MET (Figure 5).

### 2.5. Oxidative Stress During MET Degradation by T. harzianum in the Presence of LDPE, and Zn(II) or Cu(II)

**Figure 6:**
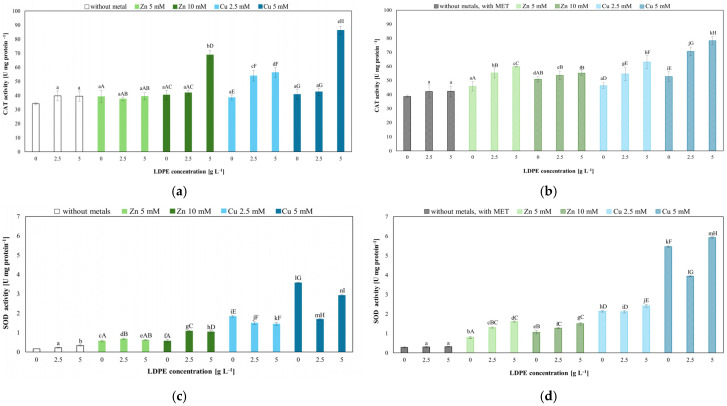
Catalase (**a**,**b**) and superoxide dismutase (**c**,**d**) activities in *T. harzianum* IM 7002 cells exposed to LDPE (2.5 and 5 g L^−1^), Zn(II) (5 and 10 mM), Cu(II) (2.5 and 5 mM), and MET (50 mg L^−1^) applied separately or in combination. Different letters indicate statistically significant differences among treatments at *p* < 0.05 between the samples (two-way ANOVA followed by post hoc Tukey’s HSD test). Lower case letters—significant effects of LDPE; uppercase letters—statistically significant effects of Zn(II) or Cu(II) concentration.

Oxidative stress was evaluated by measuring the activities of antioxidant enzymes (CAT and SOD) and lipid peroxidation (thiobarbituric acid reactive substance, TBARS). LDPE alone increased CAT activity from 34.11 (control) to 38–39 U mg^–1^ protein. The combination of LDPE (5 g L^−1^) and 10 mM of Zn(II) nearly doubled CAT activity, reaching 69.21 U mg^−1^, whereas the highest CAT activity (86.05 U mg^−1^) was observed in the presence of LDPE and 5 mM of Cu(II) (Figure 6a). MET alone caused a modest increase in CAT activity (Figure 6b, Table S2). Notably, the three-factor combination of LDPE (2.5 or 5 g L^−1^), MET, and copper (5 mM) induced the strongest enzymatic responses (80 U mg^−1^). The post hoc Tukey’s HSD test (*p* < 0.05) indicated significant differences between most treatments and the control without LDPE and Zn(II) or Cu(II). However, no significant differences were observed for Zn(II) (5 mM) combined with LDPE (2.5 g L^−1^) (Figure 6a).

SOD activity showed comparable patterns, with the strongest induction recorded in the MET + LDPE + Cu(II) treatment (Figure 6c,d, Table S3). The post hoc Tukey’s HSD test (*p* < 0.05) revealed significant differences between the treated samples and the control lacking metals and LDPE. Statistically significant differences were also observed between MET-containing samples supplemented with Zn(II) or Cu(II) and LDPE and MET-only control. However, no significant differences were detected for treatments containing LDPE at 5 g L^−1^.

**Figure 7:**
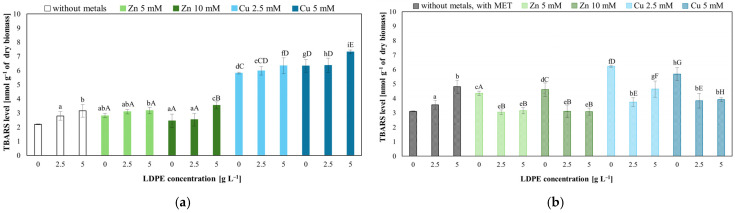
Lipid peroxidation in *T. harzianum* IM 7002 after 168 h of exposure: (**a**) cultures supplemented with LDPE and Zn(II) or Cu(II); (**b**) cultures supplemented with LDPE, Zn(II) or Cu(II) and MET. Different letters indicate statistically significant differences among treatments at *p* < 0.05 between the samples (two-way ANOVA followed by post hoc Tukey’s HSD test). Lowercase letters—significant effects of LDPE; uppercase letters—statistically significant effects of Zn(II) or Cu(II) concentration.

Elevated thiobarbituric acid reactive substance levels indicated lipid peroxidation. LDPE alone increased TBARS level in *Trichoderma* cells from 2.20 (control) to 3.0 (LDPE alone) and 4.81 nmol g^−1^ when combined with MET. Among the tested stressors, Cu(II) exhibited the strongest prooxidant effect. At a concentration of 5 mM, it caused a significant increase in TBARS. Interestingly, TBARS levels in combined MET, Zn(II), or Cu(II) and LDPE systems were lower than in the corresponding metal-only treatments (Figure 7, Table S4). Statistical analysis confirmed that most treatments differed significantly from the control without LDPE and Zn(II) or Cu(II), as well as from MET-containing systems. However, in MET-free treatments containing Zn(II) (10 mM), including the combinations of Zn(II) (10 mM) with LDPE (2.5 g L^−1^), no statistically significant differences were observed.

### 2.6. Impact of LDPE and Zn(II) or Cu(II) on Membrane Phospholipid Composition and Membrane Integrity During Metolachlor Degradation

**Figure 8:**
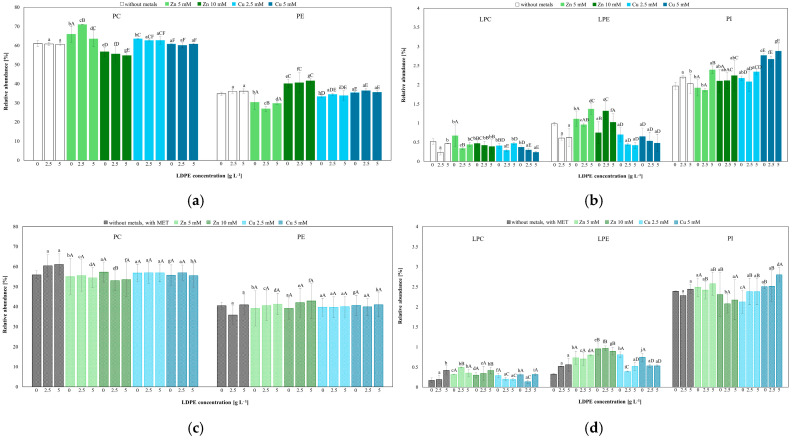
Comparison of the main phospholipid classes of *T. harzianum* IM 7002 exposed to LDPE (2.5 g L^−1^ and 5 g L^−1^), MET, and Zn(II) and Cu(II) alone or in combination: (**a**,**b**) without MET; (**c**,**d**) with MET after 7 days of incubation. Phosphatidylethanolamine (PE), phosphatidylcholine (PC), phosphatidylinositol (PI), lysophosphatidylcholine (LPC), and lysophosphatidylethanolamine (LPE). Columns marked with different letters differ significantly; *p* < 0.05 in two-way ANOVA followed by post hoc Tukey’s HSD test (lowercase letters—statistically significant effects of LDPE; uppercase letters—significant effects Zn(II) or Cu(II) concentration).

The fungal cell membrane constitutes the first barrier against adverse conditions, with phospholipids as its major structural components. The results obtained indicate that LDPE alone did not significantly affect the levels of phosphatidylcholine (PC) and phosphatidylethanolamine (PE). In MET-containing systems, LDPE addition (2.5 g L^−1^ and 5 g L^−1^) increased PC and decreased PE. Similarly, in treatments containing Zn(II) (5 mM) either alone or combined with LDPE, PC levels increased, whereas PE levels decreased. In contrast, at 10 mM Zn(II) (alone or with LDPE), the opposite changes in the PC/PE proportion were observed. In systems containing Cu(II) either alone or combined with LDPE and MET, no statistically significant effects on PC and PE levels were detected. Notably, MET addition generally decreased PC and increased PE relative to the corresponding MET-free treatments (Figure 8a,b, Table S5).

Minor phospholipid classes, such as lysophosphatidylethanolamine (LPE), lysophosphatidylcholine (LPC), and phosphatidylinositol (PI), represented only a small fraction of the total lipid pool. In LDPE-only treatments, PI increased, whereas LPC and LPE decreased. By contrast, MET increased LPC and LPE. PI decreased at 2.5 g L^−1^ LDPE and increased again at 5 g L^−1^ LDPE. In the absence of MET, lower Zn(II) and LDPE concentrations reduced PI, whereas Zn(II) (5 mM) combined with LDPE (5 g L^−1^) increased PI. Elevated PI levels were also observed in treatments containing Zn(II) (10 mM) with LDPE, as well as in Cu(II) + LDPE systems. In Zn(II)-amended systems, MET induced effects opposite to those observed in the corresponding MET-free variants. Furthermore, lower Zn(II) concentrations, alone or in combination with LDPE, increased LPC and decreased LPE, whereas higher Zn(II) concentrations elicited the opposite pattern.

A similar pattern, with a particular increase in PI, was observed in Cu(II) + LDPE + MET treatments, as well as in Cu(II) + LDPE systems without MET. Cu(II), alone or combined with LDPE, reduced LPC and LPE, whereas MET + LDPE + Zn(II)/Cu(II) treatments elicited an increase in LPC and LPE levels (Figure 8c,d).

Confocal microscopy and propidium iodide staining were applied to visualize membrane permeability changes in *T. harzianum* mycelium exposed to Zn(II) or Cu(II), LDPE, and MET, either alone or in combination. Propidium iodide penetrated only through the damaged cell membrane.

**Figure 9:**
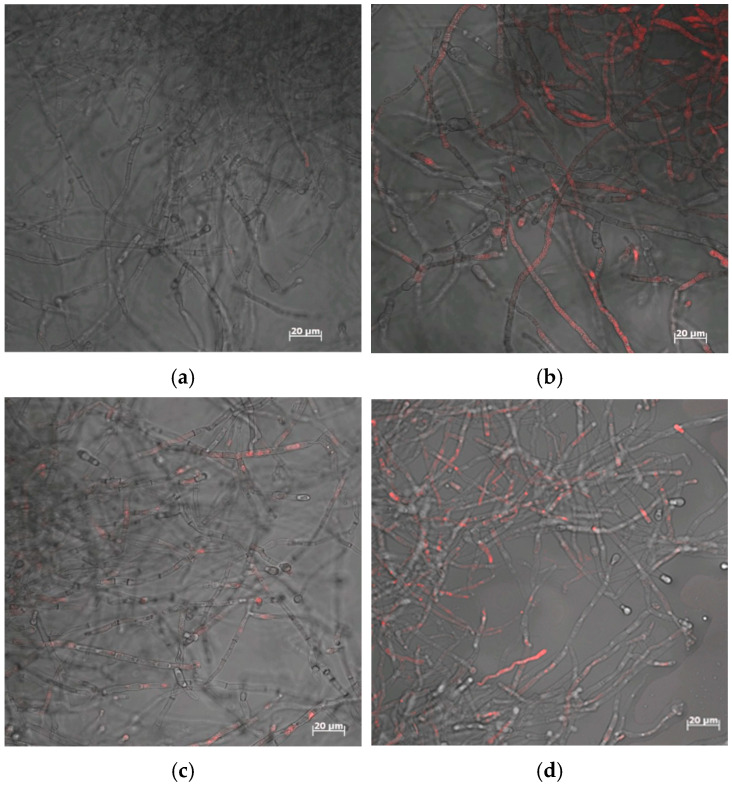
Effect of LDPE (5 g L^−1^) and/or Cu(II) (5 mM) on the permeability of *T. harzianum* cell membrane: (**a**) control without LDPE and Cu(II); (**b**) the system with LDPE; (**c**) the system with MET; (**d**) the system with Cu(II) + LDPE + MET (the red discoloration indicates the penetration of propidine iodide into the fungal hyphae).

After 7 days of incubation, increased membrane permeability was observed in *T. harzianum* IM 7002 in treatments containing LDPE, Cu(II), and MET, either alone or in combination (Figure 9). In the presence of LDPE (5 g L^−1^), Cu(II) (5 mM), MET, or their combination, membrane permeability reached 1.384%, 1.632%, 2.623%, and 4.131%, respectively, whereas no permeability was detected in the control (0.000%). In contrast, only a slight increase in membrane permeability was recorded in Zn(II)-amended systems containing LDPE and MET (Table 1).

Statistical analysis revealed significant differences (*p* < 0.05) between most treatments and the control lacking Zn(II) or Cu(II), LDPE, and MET. However, such differences were not observed for Zn(II) combined with LDPE. Significant differences (*p* < 0.05) were also confirmed between the tested systems and the MET-containing control (without Zn(II) or Cu(II) and LDPE), except for treatments containing LDPE (5 g L^−1^) and Cu(II) (5 mM), as well as those containing LDPE (2.5 g L^−1^) (Table 1 and Table S6).

Two-way ANOVA revealed that both LDPE and Zn(II) or Cu(II) significantly affected fungal physiology, including biomass, ergosterol content, antioxidant enzyme activities, lipid peroxidation, membrane permeability, and phospholipid composition (*p* < 0.05) (Figure 5, Figure 6, Figure 7 and Figure 8, Table 1). Significant interaction effects were also observed for several parameters, indicating that the combined presence of LDPE and Zn(II) or Cu(II) modulated the fungal response differently than each factor alone. The effect of MET was evaluated separately using one-way ANOVA by comparing each system with MET to the corresponding system without MET (Tables S1–S6). MET significantly affected most measured parameters (*p* < 0.05), and in some cases amplified or mitigated the effects of LDPE and metal, highlighting its role as an additional chemical stressor. Overall, these results demonstrate that both single and combined environmental stressors have distinct and sometimes synergistic effects on *T. harzianum* physiology.

## 3. Discussion

MPs contamination, together with persistent herbicides and heavy metals, poses complex stressors to soil microbial communities. MPs enter soil through agricultural film degradation, fertilizers, and sewage sludge, altering physicochemical properties and interacting with co-occurring contaminants [20,21]. Understanding the combined impact of these stressors on agriculturally relevant fungi of the genus *Trichoderma* is important for assessing soil resilience and bioremediation potential.

For a proper understanding of the effects of heavy metals on soil microorganisms, it is crucial to relate experimental conditions to actual soil concentrations of these elements. Literature data indicate that the total zinc content in agricultural soils usually ranges from approximately 10 to 300 mg kg^−1^ (0.153–4.59 mM kg^−1^) of dry soil, depending on soil type and geochemical conditions [22,23]. Total copper content in arable soils typically falls within 2 to 121.52 mg kg^−1^ (0.032–1.9 mM kg^−1^) [23,24]. Maximum allowable concentrations of copper and zinc in agricultural soils of 100 and 300 mg kg^−1^ of dry soil, respectively, are derived from current Polish regulations for assessing soil contamination [25]

Higher concentrations were applied to evaluate the *Trichoderma* strain’s tolerance and herbicide degradation potential under extreme co-contaminated conditions. Such extreme levels can occur locally in soils affected by long-term metal inputs (e.g., industrial emissions or repeated Cu-based plant protection treatments), where total soil Cu may reach very high concentrations.

*T. harzianum* IM 7002, isolated from a highly contaminated industrial landfill, showed remarkable tolerance to LDPE, Zn(II), Cu(II), and MET. Long-term exposure to complex mixtures of toxic compounds likely imposed selective pressure, favoring microorganisms with enhanced metal tolerance and well-developed enzymatic systems involved in xenobiotic detoxification. This strain efficiently degraded MET (up to 90%) even in the presence of LDPE and Zn(II) or Cu(II), suggesting adaptive metabolic pathways induced by long-term contaminant exposure.

Despite this tolerance, LDPE and MET were found to reduce fungal growth. LDPE acted as a physical and indirect chemical stressor, limiting biomass accumulation while increasing the ergosterol content. Interactions between microplastic particles and fungal hyphae may disrupt cell surfaces and induce mechanical stress, as previously reported for *T. harzianum* exposed to LDPE [26,27]. While MPs are largely chemically inert, they can release additives, residual monomers, or adsorbed contaminants that may affect soil fungi [28]. Some studies report that reduced fungal biomass under MPs exposure, while others describe increased biomass despite signs of oxidative stress, such as lipid peroxidation and increased membrane permeability [26,29,30,31]. Direct stimulation of ergosterol biosynthesis by LDPE has not been demonstrated. Therefore, the elevated ergosterol levels observed in *T. harzianum* IM 7002 are more likely an adaptive response to membrane stress and oxidative imbalance rather than a direct effect of plastic particles on sterol metabolism.

MET inhibited fungal growth, probably due to its lipophilic nature and accumulation in cellular membranes [7]. This inhibitory effect increased in the presence of LDPE, suggesting that LDPE may enhance MET bioavailability. Copper ions strongly suppressed mycelial growth and reduced the ergosterol content, whereas Zn(II) showed stimulatory effects under certain conditions. These findings support higher tolerance of *T. harzianum* to Zn(II) than to Cu(II), which is consistent with previous studies [32].

This differential response reflects the essential physiological role of zinc and the existence of efficient Zn(II) homeostatic mechanisms, in contrast to Cu(II), which, despite being also a microelement, readily induces oxidative stress, disrupts membrane integrity, and interferes with ergosterol biosynthesis [33].

This strain demonstrated a high MET degradation efficiency (up to 90%), exceeding values previously reported for *T. harzianum* [18,26]. Bernat et al. [7] reported that *T. harzianum* showed the ability to degrade the herbicide MET in the soil, and the presence of MPs favored herbicide removal. In liquid cultures of *T. harzianum* IM 7002, low LDPE concentrations did not impair MET degradation, whereas higher LDPE addition (5 g L^−1^) inhibited this process, likely due to excessive stress and metabolic disruption. Low concentrations of Zn(II) and Cu(II) stimulated fungal MET degradation, consistent with their role as micronutrients required for enzymatic activity [34,35], while higher metal concentrations exerted inhibitory effects. Interestingly, the presence of LDPE diminished MET degradation in metal-containing systems, except for high Cu(II) concentrations, where LDPE appeared to enhance degradation. It is possible that the observed process results from the generation of reactive oxygen species (ROS), which could support the transformation of MET adsorbed into plastic particles.

MET degradation was accompanied by the formation of characteristic metabolites (MDES, M2H, MOXA), resulting from oxidative reactions such as hydroxylation, side-chain oxidation, and partial dechlorination. It should be emphasized that the degradation products of metolachlor, such as MOXA, exhibit markedly lower toxicity compared to MET [36]. The toxicity of M2H and MDES has not been experimentally determined; however, based on their chemical structures, it can be expected that their toxicity is lower than that of MET, as one derivative is dechlorinated and the other hydroxylated.

These transformation products are consistent with redox-dependent enzymatic pathways and suggest that reactive oxygen species generated under oxidative stress may contribute to MET biotransformation [7,19].

Indeed, oxidative stress may be the main response of *T. harzianum* IM 7002 to LDPE, MET, and Zn(II) or Cu(II). LDPE alone has been found to increase CAT and SOD activity, and this effect is further enhanced by MET and metal ions, which may indicate increased production of ROS. Similar responses have been documented for fungi and other organisms exposed to MPs and metal–plastic mixtures [28,37,38]. Cu(II) combined with LDPE can cause strong oxidative stress, reflected in altered antioxidant enzyme activities and elevated lipid peroxidation [39].

The results obtained indicate that LDPE increases lipid peroxidation in *T. harzianum* cells, especially in combination with MET. This may be related to the fact that the excess of ROS may not be fully neutralized by the antioxidant system. Hamed et al. [40] and Barboza et al. [41] described that MPs increased lipid peroxidation in the fish, *Oreochromis niloticus* and *Dicentrarchus labrax*. Jasinska et al. [26] showed that adding MET to *Trichoderma* cultures containing MPs could enhance the lipid peroxidation process compared to a system containing MPs alone. The presence of MET increased lipid oxidation, and Cu(II) showed a stronger prooxidative effect than Zn(II). The simultaneous action of LDPE and Zn(II) or Cu(II) only enhanced this effect. Unexpectedly, the combination of LDPE, Zn(II) or Cu(II) and MET resulted in a decrease in lipid peroxidation compared to systems without MET. This may reflect redirection of ROS toward MET biotransformation rather than membrane damage. This interpretation is plausible and consistent with the simultaneous increase in degradation efficiency, suggesting a possible functional trade-off between oxidative damage and xenobiotic metabolism.

The highest levels of lipid peroxidation were found in systems containing Cu(II) and LDPE. It has been reported in the literature that heavy metals (As, Cd, Pb, Zn) in combination with microplastics increased lipid peroxidation, which was evidenced by the increase in the level of malondialdehyde [42,43,44]. Also, high concentrations of Cu(II) and combinations of MPs and Cu^2+^ can lead to increased lipid peroxidation and damage to cell membranes, indicating a possible combined effect [45,46].

The degradation of MET by *T. harzianum* IM 7002 in the presence of LDPE and Zn(II) or Cu(II) may cause oxidative stress, which was reflected in alterations of cell membrane permeability and phospholipid composition. LDPE alone slightly increased membrane permeability. Jasińska et al. [26] observed that MPs lead to increased membrane permeability in *T. harzianum* mycelia within 24 h of exposure. The literature data indicate that polyethylene (PE) microplastics may interact directly with lipid bilayers, penetrate cell membranes, and disturb their structural integrity [47,48]. Such interactions likely result in membrane stretching and destabilization, thereby increasing permeability.

The addition of MET significantly enhanced membrane permeability, which corroborates previous findings in algae (*Scenedesmus obliquus*) and higher plants, where MET exposure resulted in increased membrane leakage [49,50]. This effect is most likely associated with MET-induced ROS generation, leading to lipid peroxidation and disruption of membrane integrity [26]. In contrast, Zn(II) alone did not markedly affect membrane permeability, which agrees with a study on the stabilizing role of Zn(II) in plant cell membranes [51]. However, the combined action of MET, LDPE, and Zn(II) caused a slight increase in permeability, suggesting that under combined stress conditions, its protective effect may be insufficient to counteract oxidative damage. Cu(II) exposure clearly increased membrane permeability, and this effect was further intensified in the presence of MET and LDPE. Similar observations were reported for maize membranes and fungal cells, where Cu(II)-induced ROS generation led to reduced membrane integrity and increased permeability [34,52].

Changes in membrane permeability were accompanied by alterations in the phospholipid profile, particularly in the balance between PC and PE, which are the dominant phospholipids in *T. harzianum* membranes [53]. LDPE alone and in combination with MET caused only minor changes in the PC/PE ratio, indicating a limited impact on membrane organization. In contrast, the combined exposure to MET, Zn(II), and LDPE resulted in a decrease in PC and an increase in PE, suggesting enhanced membrane stress and increased membrane fluidity. A reduction in the PC-to-PE ratio was associated with decreased membrane rigidity and impaired membrane function under stress conditions [53].

Interestingly, the effect of Zn(II) on phospholipid composition was concentration dependent. At 5 mM of Zn(II) in the presence of LDPE, an increase in PC accompanied by a decrease in PE was observed, which may reflect the activation of adaptive mechanisms aimed at membrane stabilization. Such a shift towards PC synthesis is consistent with the role of PC in maintaining bilayer stability under moderate stress. In contrast, at 10 mM Zn(II) combined with LDPE, PC levels decreased while PE accumulated, which may result from the inhibition of PE conversion to PC and the preferential accumulation of PE under conditions of elevated oxidative stress. This interpretation is supported by reports indicating that oxidative stress can impair phospholipid remodeling pathways and favor PE accumulation.

Particularly important for the interpretation of membrane damage and remodeling is the observed alteration in lysophospholipid levels. An increase in LPE was detected in Zn(II)-treated cultures. Under physiological conditions, lysophospholipids are maintained at low levels due to the high activity of lysoacyltransferases, which rapidly reacylate them to form intact phospholipids [7]. An accumulation of lysophospholipids is therefore commonly regarded as a marker of membrane lipid hydrolysis and impaired phospholipid turnover. The increased lysophospholipid content observed under Zn(II) stress may indicate enhanced phospholipase activity and partial inhibition of lipid reacylation, reflecting membrane remodeling processes aimed at adaptation to metal-induced stress. Similar stress-induced accumulation of lysophospholipids, particularly LPE, has been reported in *Yersinia pseudotuberculosis* cells exposed to heat shock [54].

Overall, the changes observed in phospholipid and lysophospholipid levels indicate that combined exposure to LDPE, MET, and Zn(II) or Cu(II) not only induces membrane damage but also activates specific lipid remodeling pathways that reflect both the magnitude of stress impact and adaptive capacity of *T. harzianum* IM 7002.

## 4. Materials and Methods

### 4.1. Microplastics

Three LDPE fractions, differing in particle size, were used in the following proportions: ≤400 μm (20%), 500 μm (40%), and 1000 μm (40% of the total LDPE mass). The materials were purchased from Alfa Aesar (Haverhill, MA, USA). LDPE portions of 50 mg and 100 mg in Eppendorf tubes were sterilized in a Koch apparatus for 60 min.

### 4.2. Strain and Experimental Design

*T. harzianum* IM 7002 was isolated from a soil sample collected at the site of the former “Boruta” Dyeing Industry Plant in Zgierz and was included in the Collection of Microorganisms of the University of Lodz, Department of Industrial Microbiology and Biotechnology. Fungal spores used for experimental culture were obtained from 7-day cultures on ZT agar slants, the composition of which was described by Bernat et al. [7]. To initiate pre-cultures, *T. harzianum* IM 7002 spores were introduced into 100 mL Erlenmeyer flasks containing mineral Lobos medium supplemented with glucose (2%) and yeast extract (1%), to obtain a spore suspension with a density of 5 × 10^7^ mL^−1^, which was then incubated on a rotary shaker (120 rpm) at 28 °C for 24 h. In biotic control cultures, 2 mL of pre-culture was added to 18 mL of fresh medium. In selected experimental variants, LDPE (2.5 or 5 g L^−1^), MET at a concentration of 50 mg L^−1^, and ZnSO_4_ × 7H_2_O (5 or 10 mM), or CuSO_4_ × 5H_2_O (2.5 or 5 mM) were added to the culture. MET and the metal salts were purchased from Sigma-Aldrich, Merck, Darmstadt, Germany. Appropriate biotic and abiotic controls were prepared for all experimental variants. Cultures were incubated for 7 days on a rotary shaker (120 rpm) at 28 °C, after which the samples were subjected to further analyses.

The experiment was designed as a completely randomized design using a multifactorial approach to evaluate the main and interaction effects of LDPE and heavy metals (Zn(II) or Cu(II)) in the presence or absence of MET. Zn(II) and Cu(II) effects were analyzed in separate experimental series and were not combined within the same experimental system. The study was conducted in three stages:

(i) Preliminary dose–response screening to assess fungal spores’ viability and mycelia tolerance to metals;
(ii) MET degradation factorial experiments, metal concentration × MP concentration (2.5, 5 g L^−1^), performed separately for Zn(II) (5 and 10 mM) and Cu(II) (2.5 and 5 mM), resulting in a total of 15 systems;
(iii) Assessment of fungal physiological and biochemical responses in 15 factorial systems without MET and 15 systems with MET, to evaluate the combined effects of MET, Cu(II)/Zn(II), and LDPE.

### 4.3. Molecular Identification of T. harzianum IM 7002

#### 4.3.1. DNA Extraction

DNA was isolated from the *Trichoderma* isolate exhibiting promising MET degradation potential. The strain was cultivated on a ZT agar plate for 168 h. Mycelium was harvested, and the DNA was extracted using the GeneMATRIX Plant& Fungi DNA Purification Kit (EURx, Gdansk, Poland). The isolated DNA was stored at −20 °C.

#### 4.3.2. PCR and DNA Sequencing of *T. harzianum* IM 7002

PCR amplification of the ITS region was performed using primers ITS1F (CTT-GGT CAT-TTA-GAG-GAA-GTA-A) and ITS4 (TCC-TCC-GCT-TAT-TGA-TAT-GC) with OptiTaq DNA Polymerase (EURx, Gdansk, Poland). The 25 μL reaction mixture contained: 15.25 μL distilled water, 2.5 μL of 10 × Pol Buffer B, 1 μL of 0.1 mM dNTP mix (5 mM each), 2 μL of 100 mmol primers ITS1 and ITS4, 0.25 μL of 1.25 U OptiTaq DNA Polymerase, and 2 μL of DNA template. The PCR conditions were as follows: initial denaturation stage at 95 °C for 4 min, followed by 35 cycles at 95 °C for 30 s, 54 °C for 30 s, 72 °C for 90 s, and then final elongation at 72 °C for 10 min. PCR products were purified and sequenced by Genomed, Warsaw, Poland.

#### 4.3.3. Phylogenetic Placement of *T. harzianum* IM 7002

The ITS sequence obtained from the isolate was queried against the NCBI nucleotide database [55] using the BLAST 2.13.0+ algorithm [56] to assign an initial taxonomic position. To determine the exact phylogenetic position of the strain, ITS sequences from *Trichoderma atroviride*, *T. harzianum*, and *T. citrinoviride* were downloaded from the NCBI database. Additionally, ITS sequences from *Metarhizium eburneum* and *M. brachyspermum* were included as outgroup references. The sequences were aligned using the MAFFT v7.407 program [57] and trimmed using trimAl v1.4.rev22 [58]. Modeltest-ng v0.1.7 [59] was used to determine the best model of evolution for the dataset. A phylogenetic tree was constructed using IQ-TREE v1.6.9 [60] with 1000 standard non-parametric bootstrap replicates and a maximum likelihood tree search. It was then visualized using iTOL v5 [61].

### 4.4. Spore Viability and Metal Tolerance of T. harzianum IM 7002

Three types of samples were prepared in this experiment: abiotic controls, biotic controls, and test samples. The following were applied to the titer plates: 100 μL of Lobos medium with 2% glucose (abiotic assays), 50 μL of substrate and 50 μL of spore suspension (5 × 10^7^ mL^−1^) in biotic assays, and 50 μL of substrate and 50 μL of the test compound solution (MET, 10 mg mL^−1^, Cu(II) or Zn(II) at a concentration of 40 mM L^−1^) together with 50 μL of spore suspension. The plates were incubated at 28 °C for 72 h. Then 150 μL of fluorescein diacetate solution (2 mg mL^−1^ from Sigma-Aldrich, Darmstadt, Germany, in 0.1 M phosphate buffer from Chempur, Piekary Śląskie, Poland) was added to each well and incubated in the dark for 2 h. Fluorescence was measured at excitation/emission wavelengths of 485/520 nm using a FLUOstar Omega version 5.10 R2 software (BMG Labtech, Ortenberg, Germany) reader.

An appropriate volume of metal salt solutions was added to the Lobos liquid medium containing glucose (2%) and yeast extract (1%) to achieve the required concentrations: 1.0, 2.5, 5.0, 7.5, and 10 mM. Then, 10% of fungal pre-cultures were introduced into the flasks, and the cultures were incubated for 7 days on a rotary shaker at 120 rpm and 28 °C. After incubation, the biomass was filtered using a Rocker 300C PTFE vacuum pump (Rocker Scientific Co., Ltd., Kaohsiung City, Taiwan) through Whatman No. 1 filter papers (Merck KGaA, Darmstadt, Germany) and then thoroughly washed with deionized water. The collected mycelium was dried in an oven at 100 °C, and the dry matter was weighed using an electronic balance.

### 4.5. Analysis of Metolachlor Content

Seven-day-old cultures of *T. harzianum* IM 7002 exposed to herbicide were extracted using the QuEChERS method. A mixture of salts (2 g MgSO_4_, 0.5 g NaCl, 0.5 g C_6_H_5_Na_3_O_7_ × 2H_2_O, 0.25 g C_6_H_5_Na_3_O_7_ × 1.5H_2_O, purchased from Merck, Darmstadt, Germany) was added to Falcon tubes, followed by 10 mL of acetonitrile (Merck, Darmstadt, Germany) and the fungal cultures. The samples were homogenized with glass beads using a Ball Mill MM 400 ball homogenizer (Retsch, Haan, Germany) for 5 min at 30 Hz and then centrifuged at 2000× *g* for 5 min. One milliliter of the supernatants was transferred to Eppendorf tubes, and then the obtained extracts were diluted with a water–methanol mixture (80:20, *v*/*v*).

Chromatographic analyses were performed using an Agilent 1200 Series HPLC system (Agilent Technologies, Santa Clara, CA, USA) coupled with a mass detector (Sciex, Framingham, MA, USA) equipped with an ESI. A 10 μL aliquot of the sample was injected into a Kinetex C18 column (50 × 2.1 mm, 5 μm; Phenomenex, Torrance, CA, USA) maintained at 35 °C with a flow rate of 0.5 mL min^−1^. The mobile phases consisted of water (A) and methanol (B), both containing 5 mM ammonium formate. The gradient program was as follows: from 80% A (1 min) to 10% A (1 min), maintaining 10% A (2.5 min), returning to the initial conditions (2 min). Ionization was performed in positive mode at 500 °C. For the MET analysis, the multiple reaction monitoring (MRM) transitions were *m*/*z* 284.09–252.2 and 284.09–176.3. To identify possible degradation metabolites, the EMS→EPI information-dependent acquisition method was applied. Based on product ion analysis, MRM transitions were established for M2H (*m*/*z* 266.1→176 and 266.1→234.1) and MDES (*m*/*z* 250.1→176.1 and 250.1→218.1). To ensure that the loss of MET was due to fungal activity and not adsorption of MET onto the LDPE surface, appropriate abiotic controls were included, and the MET recovery percentages were calculated (Table S7, Supplementary Materials).

### 4.6. Enzyme Activity Determination

After incubation of the culture, 300 mg of fresh mycelium was collected and homogenized in a frozen mortar with 3 mL of 50 mM sodium phosphate buffer (pH 7), whose composition was described by Jasińska et al. [26]. The homogenate was transferred to 2 mL of Eppendorf tubes and centrifuged at 15,000× *g* for 10 min at 4 °C. using an MPW 260RH centrifuge (MPW Med. Instruments, Warsaw, Poland). The supernatant was then collected and stored on ice until analysis. Reagents for SOD and CAT activity assays were purchased from Merck, Darmstadt, Germany. SOD activity was measured spectrophotometrically based on reduction in nitrotetrazole blue chloride at 540 nm, while CAT activity was determined by monitoring the decomposition of hydrogen peroxide at 240 nm. SOD and CAT activities were expressed as units per milligram of protein, and the protein content was determined using the Bradford method (Bradford reagent from Chempur, Piekary Śląskie, Poland).

### 4.7. Determination of Phospholipids and Ergosterol

After 7 days of incubation, 50 mg of fungal biomass was weighed and transferred to a 2 mL Eppendorf tube, followed by the addition of 1 mL of methanol. Samples were homogenized with glass beads using a Ball Mill MM 400 homogenizer (Retsch, Haan, Germany) for 4 min and then centrifuged at 10,000× *g* for 5 min. The supernatant was collected into Eppendorf tubes and used for ergosterol and phospholipid analysis.

Ergosterol content was determined using a QTRAP 3200 spectrometer (Sciex) coupled with a 1200 series HPLC system (Agilent, USA). Fractionation was performed on a Kinetex C18 column (50 mm × 2.1 mm, particle size: 5 μm; Phenomenex, Torrance, CA, USA) using a gradient with water (A) and methanol (B) as mobile phases, both with 5 mM ammonium formate. The analytes were separated using a gradient: from 60% solvent A (0–1 min), then increasing the content of solvent B to 100% (1–4 min), then returning to 60% solvent A from 4.0 to 4.1 min and maintaining 60% until 6 min; flow rate was 0.8 mL min^−1^. Mass spectrometric detection was carried out in positive ion mode with an atmospheric pressure chemical ionizer (APCI) source at 550 °C. The analysis was performed using multiple reaction monitoring (MRM) with the following transitions: *m*/*z* 379.3–69.1 and 379.3–81.3 for ergosterol; and *m*/*z* 369.3–147.2 for cholesterol, which was used as the internal standard.

Phospholipids were isolated from *T. harzianum* IM 7002 biomass according to the method described by Bernat et al. [7,62]. Lipid extract was separated using an Exion LC AC UHPLC system (Sciex, Framingham, MA, USA). The mobile phase was the same as that used for ergosterol analysis. Separation was performed on a Kinetex C18 column (50 mm × 2.1 mm, particle size: 5 μm; Phenomenex, Torrance, CA, USA) at 40 °C, at a flow rate of 0.5 mL min^−1^. The gradient program was as follows: from 30% A for 0.25 min, increasing to 95% B (1 min), maintaining 95% B for 5 min, then returning to the initial conditions (2 min). Mass spectrometry parameters were as follows: spray voltage 4500 V, curtain gas 25 psi, nebulizer 60 psi, turbo gas 50 psi, and source temperature 600 °C. Data were analyzed using Analyst™ v1.6.3 software (Sciex, Framingham, MA, USA). Qualitative and quantitative analyses were performed as described by Bernat et al. [7].

### 4.8. Lipid Peroxidation Assay

The degree of lipid peroxidation was assessed based on the content of TBARS, according to a modified method described by Jo and Ahn [63] and Bernat et al. [52]. Freshly harvested culture was transferred to a 2 mL Eppendorf tube and centrifuged at 10,000× *g* for 10 min using an MPW 260RH centrifuge (MPW Med. Instruments, Warsaw, Poland). A 200 μL aliquot of supernatant was transferred to a new Eppendorf tube, and 400 μL of TBA–TCA solution (20 mM TBA from Sigma, Darmstadt, Germany, in 15% TCA from Chempur, Piekary Śląskie, Poland) was added. The mixture was thoroughly vortexed, incubated in a water bath at 95 °C for 30 min, cooled in a cold water bath for 10 min, and then centrifuged for 10 min at 10,000× *g*. Absorbance of the supernatant was measured spectrophotometrically at 531 nm using a FLUOstar Omega (BMG Labtech, Ortenberg, Germany), and corrected for non-specific absorption at 600 nm.

### 4.9. Membrane Permeability

Membrane permeability was assessed using propidium iodide, according to the method described by Jasińska et al. [26] with modifications. Mycelium collected from 1 mL of culture was centrifuged (6 min, 6000× *g*) and then suspended in 1 mL of PBS buffer (pH 7, Sigma, Darmstadt, Germany). Ten microliters of propidium iodide solution (1 mg mL^−1^, Sigma, Darmstadt, Germany) were added, and the samples were incubated in the dark at room temperature for 5 min. After incubation, the sample was centrifuged again (5 min, 8000× *g*), the supernatant was removed, and the mycelium was washed twice with PBS. It was then suspended in 0.5 mL of PBS and transferred to a slide for observation under the Nikon Eclipse E100 light microscope (Nikon Corporation, Tokyo, Japan). The results are expressed as a percentage of mycelium stained with propidium iodide, representing dead cells relative to total mycelium in relation to the entire mycelium.

### 4.10. Statistical Analysis

The experiment data represent the mean values obtained from three independent biological replicates ± standard deviation (SD), *n* = 3. Each biological replicate corresponded to an independently prepared culture. Statistical analyses of the data were carried out using the Statistica software (13.1). Data was analyzed using a one-way ANOVA was performed, followed by Dunnett’s test for comparisons relative to control samples and two-way analysis of variance (ANOVA) using post hoc Tukey’s HSD test, to determine the significance of the differences at *p* < 0.05. The effect of MET was assessed separately by comparing systems with and without MET using one-way ANOVA followed by Tukey’s HSD test. The charts were created using Excel software for Microsoft 365.

## 5. Conclusions

This study demonstrated that the *Trichoderma harzianum* IM 7002 strain, isolated from a heavily polluted environment, effectively degrades metolachlor both alone and in the presence of a heavy metal (Zn(II) or Cu(II)) and LDPE. Lower concentrations of Zn(II)) (5 mM) and Cu(II) 2.5 mM enhanced MET degradation, whereas higher concentrations, 10 mM Zn(II) and 5 mM Cu(II), partially inhibited this process. LDPE influenced the effect of metals on degradation, amplifying the inhibitory effect of Zn(II) (10 mM), while mitigating the negative effect of Cu(II) (5 mM).

Exposure to Zn(II) or Cu(II) and LDPE induced changes in phospholipid profiles and a slight increase in membrane permeability, indicating its partial destabilization, while increased activities of antioxidant enzymes CAT and SOD reflected activation of cellular defense mechanisms. Interestingly, samples containing LDPE, MET, and metal ions exhibited lower lipid peroxidation, suggesting that reactive oxygen species may be partially consumed during MET degradation. However, causal relationships require further investigation. This work provides important insights into the degradation potential of fungi in agricultural soils contaminated with LDPE, MET, Zn(II), or Cu(II), contributing to a better understanding of their role in pollutant removal. Additionally, changes in the bioavailability of metals or herbicides due to adsorption–desorption processes on LDPE, as well as direct interactions of metal ions with enzymes involved in the degradation pathway, are likely important factors influencing the observed modulation and will be addressed in future studies.

## Figures and Tables

**Table 1:** Effect of LDPE (2.5 g L^−1^ or 5 g L^−1^), Zn(II) or Cu(II), and MET (50 mg L^−1^) on *T. harzianum* IM 7002 cell membrane permeability (expressed as a percentage representing the ratio of propidine-iodide-stained mycelium, i.e., dead, to the entire mycelium) after 7 days of culture. The different letters indicate a significant difference (*p* < 0.05, two-way ANOVA followed by post hoc Tukey’s HSD test). Lowercase letters—significant effect of LDPE; uppercase letters—statistically significant effect of Zn(II) or Cu(II).

Culture	Membrane Permeability [%]	
	Without Metolachlor	with Metolachlor
control	0.000 ±0.000	2.623 ± 0.010
LDPE 2.5 g L^−1^	1.224 ± 0.046 ^a^	1.560 ± 0.030 ^a^
LDPE 5 g L^−1^	1.384 ± 0.030 ^a^	2.583 ± 0.035 ^b^
Zn 5 mM	0.000 ±0.000 ^bA^	0.635 ± 0.021 ^cA^
Zn 5 mM + LDPE 2.5 g L^−1^	0.000 ± 0.000 ^cA^	0.831 ± 0.014 ^dA^
Zn 5 mM + LDPE 5 g L^−1^	0.008 ± 0.001 ^dA^	0.957 ± 0.017 ^eA^
Zn 10 mM	0.000 ±0.000 ^eA^	0.643 ± 0.024 ^fA^
Zn 10 mM + LDPE 2.5 g L^−1^	0.160 ± 0.030 ^fA^	0.904 ± 0.005 ^gA^
Zn 10 mM + LDPE 5 g L^−1^	0.172 ± 0.002 ^gA^	1.005 ± 0.012 ^aA^
Cu 2.5 mM	1.482 ± 0.041 ^aB^	1.713 ± 0.009 ^aB^
Cu 2.5 mM + LDPE 2.5 g L^−1^	1.668 ± 0.04 ^aB^	2.188 ± 0.059 ^aBC^
Cu 2.5 mM + LDPE 5 g L^−1^	2.406 ± 0.018 ^hC^	3.120 ± 0.065 ^hD^
Cu 5 mM	1.632 ± 0.070 ^aB^	1.725 ± 0.03 ^aBE^
Cu 5 mM + LDPE 2.5 g L^−1^	2.230 ± 0.051 ^iC^	2.453 ± 0.05 ^bCE^
Cu 5 mM + LDPE 5 g L^−1^	2.572 ± 0.114 ^jC^	4.131 ± 0.04 ^iF^

## Data Availability

The original contributions presented in this study are included in the article/Supplementary Material. Further inquiries can be directed to the corresponding author.

## Supplementary Materials

The following supporting information can be downloaded at: https://www.mdpi.com/article/10.3390/molecules31061038/s1, Table S1: statistical differences in the dry biomass of *T. harzianum* with or without MET; Table S2: statistical differences in the catalase activity of *T. harzianum* IM 7002 with or without MET; Table S3: statistical differences in the superoxide dismutase activity of *T. harzianum* IM 7002 with or without MET; Table S4: statistical differences in the lipid peroxidation of *T. harzianum* IM 7002 with or without MET; Table S5: statistical differences in the major phospholipid classes of *T. harzianum* IM 7002 with or without MET; Table S6: statistical differences in the cell membrane permeability of *T. harzianum* IM 7002 with or without MET; Table S7: Extraction efficiency of MET using the QuEChERS method under different abiotic control variants.

## Abbreviations

The following abbreviations are used in this manuscript:

MPs	Microplastics
MET	Metolachlor
LDPE	Low-Density Polyethylene
ROS	Reactive Oxygen Species
MDES	Metolachlor deschloro
M2H	Metolachlor-2-hydroxy
MOXA	Metolachlor oxanilic acid
CAT	Catalase
SOD	Superoxide dismutase
TIBARS	Thiobarbituric acid reactive substances
PE	Phosphatidylethanolamine
PC	Phosphatidylcholine
PI	Phosphatidylinositol
LPC	Lysophosphatidylcholine
LPE	Lysophosphatidylethanolamine
PE	Polyethylene
