# Supplementary material for: Combined Effects of Low-Density Polyethylene (LDPE), Zn(II), Cu(II), and Metolachlor on Trichoderma harzianum Growth, Oxidative Stress Induction, and Herbicide Degradation
Source: Molecules. 2026 Mar 20;31(6):1038. doi: 10.3390/molecules31061038 (PMC13028794; doi:10.3390/molecules31061038)
Supplement: Supplementary file 1 [file molecules-31-01038-s001.zip › molecules-4169249-supplementary.pdf]

Supplementary Materials

Table S1: statistical differences in the dry biomass of *Trichoderma harzianum* with or without metolachlor (MET) ($p < 0.05$, one-way ANOVA followed by post-hoc Tukey's HSD test). LDPE – low-density polyethylene.

No.	Sample	p-value
1.	without MET vs MET	<0.0001
2.	without MET + LDPE 2.5 g L^{-1} vs MET + LDPE 2.5 g L^{-1}	<0.0001
3	without MET + LDPE 5 g L^{-1} vs MET + LDPE 5 g L^{-1}	<0.0001
4.	without MET + Zn 5 mM vs MET+ Zn 5 mM	<0.0001
5.	without MET + Zn 5 mM + LDPE 2.5 g L^{-1} vs MET + Zn 5 mM + LDPE 2.5 g L^{-1}	<0.0001
6.	without MET + Zn 5 mM + LDPE 5 g L^{-1} vs MET + Zn 5 mM + LDPE 5 g L^{-1}	0.0760
7.	without MET + Zn 10 mM vs MET+ Zn 10 mM	<0.0001
8.	without MET + Zn 10 mM + LDPE 2.5 g L^{-1} vs MET + Zn 10 mM + LDPE 2.5 g L^{-1}	<0.0001
9.	without MET + Zn 10 mM + LDPE 5 g L^{-1} vs MET + Zn 10 mM + LDPE 5 g L^{-1}	<0.0001
10.	without MET + Cu 2.5 mM vs MET+ Cu 2.5 mM	<0.0001
11.	without MET + Cu 2.5 mM + LDPE 2.5 g L^{-1} vs MET + Cu 2.5 mM + LDPE 2.5 g L^{-1}	<0.0001
12.	without MET + Cu 2.5 mM + LDPE 5 g L^{-1} vs MET + Cu 2.5 mM + LDPE 5 g L^{-1}	<0.0001
13.	without MET + Cu 5 mM vs MET+ Cu 5 mM	<0.0001
14.	without MET + Cu 5 mM + LDPE 2.5 g L^{-1} vs MET + Cu 5 mM + LDPE 2.5 g L^{-1}	<0.0001
15.	without MET + Cu 5 mM + LDPE 5 g L^{-1} vs MET + Cu 5 mM + LDPE 5 g L^{-1}	<0.0001

Table S2: statistical differences in the catalase activity of *T. harzianum* IM 7002 with or without MET ($p < 0.05$, one-way ANOVA followed by post-hoc Tukey's HSD test).

No.	Sample	p-value
1.	without MET vs MET	0.8244
2.	without MET + LDPE 2.5 g L^{-1} vs MET + LDPE 2.5 g L^{-1}	0.5119
3	without MET + LDPE 5 g L^{-1} vs MET + LDPE 5 g L^{-1}	0.8682
4.	without MET + Zn 5 mM vs MET+ Zn 5 mM	0.1436
5.	without MET + Zn 5 mM + LDPE 2.5 g L^{-1} vs MET + Zn 5 mM + LDPE 2.5 g L^{-1}	0.0009
6.	without MET + Zn 5 mM + LDPE 5 g L^{-1} vs MET + Zn 5 mM + LDPE 5 g L^{-1}	<0.0001
7.	without MET + Zn 10 mM vs MET+ Zn 10 mM	0.0019
8.	without MET + Zn 10 mM + LDPE 2.5 g L^{-1} vs MET + Zn 10 mM + LDPE 2.5 g L^{-1}	0.0005
9.	without MET + Zn 10 mM + LDPE 5 g L^{-1} vs MET + Zn 10 mM + LDPE 5 g L^{-1}	0.0001
10.	without MET + Cu 2.5 mM vs MET+ Cu 2.5 mM	0.0228
11.	without MET + Cu 2.5 mM + LDPE 2.5 g L^{-1} vs MET + Cu 2.5 mM + LDPE 2.5 g L^{-1}	>0.9999
12.	without MET + Cu 2.5 mM + LDPE 5 g L^{-1} vs MET + Cu 2.5 mM + LDPE 5 g L^{-1}	0.0457
13.	without MET + Cu 5 mM vs MET+ Cu 5 mM	0.0011
14.	without MET + Cu 5 mM + LDPE 2.5 g L^{-1} vs MET + Cu 5 mM + LDPE 2.5 g L^{-1}	<0.0001
15.	without MET + Cu 5 mM + LDPE 5 g L^{-1} vs MET + Cu 5 mM + LDPE 5 g L^{-1}	0.0858

Table S3: statistical differences in the superoxide dismutase activity of *T. harzianum* IM 7002 with or without MET ($p < 0.05$, one-way ANOVA followed by post-hoc Tukey's HSD test).

No.	Sample	p-value
1.	without MET vs MET	0.0336
2.	without MET + LDPE 2.5 g L^{-1} vs MET + LDPE 2.5 g L^{-1}	0.2716
3	without MET + LDPE 5 g L^{-1} vs MET + LDPE 5 g L^{-1}	>0.9999
4.	without MET + Zn 5 mM vs MET+ Zn 5 mM	<0.0001
5.	without MET + Zn 5 mM + LDPE 2.5 g L^{-1} vs MET + Zn 5 mM + LDPE 2.5 g L^{-1}	<0.0001
6.	without MET + Zn 5 mM + LDPE 5 g L^{-1} vs MET + Zn 5 mM + LDPE 5 g L^{-1}	<0.0001
7.	without MET + Zn 10 mM vs MET+ Zn 10 mM	<0.0001
8.	without MET + Zn 10 mM + LDPE 2.5 g L^{-1} vs MET + Zn 10 mM + LDPE 2.5 g L^{-1}	0.0003

9.	without MET + Zn 10 mM + LDPE 5 g L^{-1} vs MET + Zn 10 mM + LDPE 5 g L^{-1}	<0.0001
10.	without MET + Cu 2.5 mM vs MET+ Cu 2.5 mM	<0.0001
11.	without MET + Cu 2.5 mM + LDPE 2.5 g L^{-1} vs MET + Cu 2.5 mM + LDPE 2.5 g L^{-1}	<0.0001
12.	without MET + Cu 2.5 mM + LDPE 5 g L^{-1} vs MET + Cu 2.5 mM + LDPE 5 g L^{-1}	<0.0001
13.	without MET + Cu 5 mM vs MET+ Cu 5 mM	<0.0001
14.	without MET + Cu 5 mM + LDPE 2.5 g L^{-1} vs MET + Cu 5 mM + LDPE 2.5 g L^{-1}	<0.0001
15.	without MET + Cu 5 mM + LDPE 5 g L^{-1} vs MET + Cu 5 mM + LDPE 5 g L^{-1}	<0.0001

Table S4: statistical differences in the lipid peroxidation of *T. harzianum* IM 7002 with or without MET ($p < 0.05$, one-way ANOVA followed by post-hoc Tukey's HSD test).

No.	Sample	*p*-value
1.	without MET vs MET	<0.0001
2.	without MET + LDPE 2.5 g L^{-1} vs MET + LDPE 2.5 g L^{-1}	<0.0001
3	without MET + LDPE 5 g L^{-1} vs MET + LDPE 5 g L^{-1}	<0.0001
4.	without MET + Zn 5 mM vs MET+ Zn 5 mM	<0.0001
5.	without MET + Zn 5 mM + LDPE 2.5 g L^{-1} vs MET + Zn 5 mM + LDPE 2.5 g L^{-1}	>0.9999
6.	without MET + Zn 5 mM + LDPE 5 g L^{-1} vs MET + Zn 5 mM + LDPE 5 g L^{-1}	0.9988
7.	without MET + Zn 10 mM vs MET+ Zn 10 mM	<0.0001
8.	without MET + Zn 10 mM + LDPE 2.5 g L^{-1} vs MET + Zn 10 mM + LDPE 2.5 g L^{-1}	<0.0001
9.	without MET + Zn 10 mM + LDPE 5 g L^{-1} vs MET + Zn 10 mM + LDPE 5 g L^{-1}	<0.0001
10.	without MET + Cu 2.5 mM vs MET+ Cu 2.5 mM	<0.0001
11.	without MET + Cu 2.5 mM + LDPE 2.5 g L^{-1} vs MET + Cu 2.5 mM + LDPE 2.5 g L^{-1}	<0.0001
12.	without MET + Cu 2.5 mM + LDPE 5 g L^{-1} vs MET + Cu 2.5 mM + LDPE 5 g L^{-1}	<0.0001
13.	without MET + Cu 5 mM vs MET+ Cu 5 mM	<0.0001
14.	without MET + Cu 5 mM + LDPE 2.5 g L^{-1} vs MET + Cu 5 mM + LDPE 2.5 g L^{-1}	<0.0001
15.	without MET + Cu 5 mM + LDPE 5 g L^{-1} vs MET + Cu 5 mM + LDPE 5 g L^{-1}	<0.0001

Table S5: statistical differences in the major phospholipid classes of *T. harzianum* IM 7002 with or without MET ($p < 0.05$, one-way ANOVA followed by post-hoc Tukey's HSD test). Phosphatidylcholine (PC), phosphatidylethanolamine (PE), lysophosphatidylcholine (LPC), lysophosphatidylethanolamine (LPE). phosphatidylinositol (PI).

No.	Sample	*p*-value				
		PC	PE	LPC	LPE	PI
1.	without MET vs MET	<0.0001	<0.0001	<0.0001	<0.0001	<0.0001
2.	without MET + LDPE 2.5 g L^{-1} vs MET + LDPE 2.5 g L^{-1}	>0.9999	0.8399	0.1451	0.1261	>0.9999
3	without MET + LDPE 5 g L^{-1} vs MET + LDPE 5 g L^{-1}	0.9998	<0.0001	0.7711	0.8427	<0.0001
4.	without MET + Zn 5 mM vs MET+ Zn 5 mM	<0.0001	<0.0001	<0.0001	<0.0001	<0.0001
5.	without MET + Zn 5 mM + LDPE 2.5 g L^{-1} vs MET + Zn 5 mM + LDPE 2.5 g L^{-1}	<0.0001	<0.0001	<0.0001	<0.0001	<0.0001
6.	without MET + Zn 5 mM + LDPE 5 g L^{-1} vs MET + Zn 5 mM + LDPE 5 g L^{-1}	<0.0001	<0.0001	0.0487	<0.0001	0.0040
7.	without MET + Zn 10 mM vs MET+ Zn 10 mM	0.2163	0.1873	0.0004	<0.0001	0.0019
8.	without MET + Zn 10 mM + LDPE 2.5 g L^{-1} vs MET + Zn 10 mM + LDPE 2.5 g L^{-1}	<0.0001	<0.0001	0.0487	<0.0001	0.9421
9.	without MET + Zn 10 mM + LDPE 5 g L^{-1} vs MET + Zn 10 mM + LDPE 5 g L^{-1}	0.0176	0.0518	0.9203	0.0006	0.7431
10.	without MET + Cu 2.5 mM vs MET+ Cu 2.5 mM	<0.0001	<0.0001	0.0020	0.3191	>0.9999
11.	without MET + Cu 2.5 mM + LDPE 2.5 g L^{-1} vs MET + Cu 2.5 mM + LDPE 2.5 g L^{-1}	<0.0001	<0.0001	0.8298	0.9415	<0.0001
12.	without MET + Cu 2.5 mM + LDPE 5 g L^{-1} vs MET + Cu 2.5 mM + LDPE 5 g L^{-1}	<0.0001	<0.0001	<0.0001	0.0893	>0.9999
13.	without MET + Cu 5 mM vs MET+ Cu 5 mM	<0.0001	<0.0001	0.2597	0.0245	0.0004

14.	without MET + Cu 5 mM + LDPE 2.5 g L^{-1} vs MET + Cu 5 mM + LDPE 2.5 g L^{-1}	<0.0001	<0.0001	<0.0001	>0.9999	0.1438
15.	without MET + Cu 5 mM + LDPE 5 g L^{-1} vs MET + Cu 5 mM + LDPE 5 g L^{-1}	<0.0001	<0.0001	0.0387	0.9616	0.9995

Table S6: statistical differences in the cell membrane permeability of *T. harzianum* IM 7002 with or without MET ($p < 0.05$, one-way ANOVA followed by post-hoc Tukey's HSD test).

No.	Sample	*p*-value
1.	without MET vs MET	<0.0001
2.	without MET + LDPE 2.5 g L^{-1} vs MET + LDPE 2.5 g L^{-1}	<0.0001
3	without MET + LDPE 5 g L^{-1} vs MET + LDPE 5 g L^{-1}	<0.0001
4.	without MET + Zn 5 mM vs MET+ Zn 5 mM	<0.0001
5.	without MET + Zn 5 mM + LDPE 2.5 g L^{-1} vs MET + Zn 5 mM + LDPE 2.5 g L^{-1}	<0.0001
6.	without MET + Zn 5 mM + LDPE 5 g L^{-1} vs MET + Zn 5 mM + LDPE 5 g L^{-1}	<0.0001
7.	without MET + Zn 10 mM vs MET+ Zn 10 mM	<0.0001
8.	without MET + Zn 10 mM + LDPE 2.5 g L^{-1} vs MET + Zn 10 mM + LDPE 2.5 g L^{-1}	<0.0001
9.	without MET + Zn 10 mM + LDPE 5 g L^{-1} vs MET + Zn 10 mM + LDPE 5 g L^{-1}	<0.0001
10.	without MET + Cu 2.5 mM vs MET+ Cu 2.5 mM	<0.0001
11.	without MET + Cu 2.5 mM + LDPE 2.5 g L^{-1} vs MET + Cu 2.5 mM + LDPE 2.5 g L^{-1}	<0.0001
12.	without MET + Cu 2.5 mM + LDPE 5 g L^{-1} vs MET + Cu 2.5 mM + LDPE 5 g L^{-1}	<0.0001
13.	without MET + Cu 5 mM vs MET+ Cu 5 mM	<0.0001
14.	without MET + Cu 5 mM + LDPE 2.5 g L^{-1} vs MET + Cu 5 mM + LDPE 2.5 g L^{-1}	<0.0001
15.	without MET + Cu 5 mM + LDPE 5 g L^{-1} vs MET + Cu 5 mM + LDPE 5 g L^{-1}	<0.0001

Table S7: Extraction efficiency of MET using the QuEChERS method under different abiotic control variants.

Sample	MET concentration [µg]	MET concentration [µg] mean±SD	% recovery	% recovery mean±SD
Medium + MET	768		76.8	
	742	840 ± 148	74.2	84.0 ± 14.8
	1012		101.2	
Medium + MET + LDPE 2.5 g L^{-1}	814		81.4	
	818	808 ± 13	81.8	80.8 ± 1.3
	793		79.3	
Medium + MET + LDPE 5 g L^{-1}	819		81.9	
	765	825 ± 63	76.5	82.5 ± 6.3
	891		89.1	
Medium + MET + Zn 5 mM	741		74.1	
	852	829 ± 79	85.2	82.9 ± 7.9
	895		89.5	
Medium + MET + Zn 5 mM LDPE 2.5 g L^{-1}	755		75.5	
	865	806 ± 55	86.5	80.6 ± 5.5
	800		80.0	
Medium + MET + Zn 5 mM LDPE 5 g L^{-1}	837		83.7	
	704	797 ± 80	70.4	79.7 ± 8.0
	850		85.0	
Medium + MET + Zn 10 mM	782		78.2	
	809	869 ± 128	80.9	86.9 ± 12.8
	1016		101.6	
Medium + MET + Zn 10 mM LDPE 2.5 g L^{-1}	819		81.9	
	755	826 ± 74	75.5	82.6 ± 7.4
	904		90.4	

Medium + MET + Zn 10 mM<br>LDPE 5 g L^{-1}	645<br>714<br>803	720 ± 79	64.5<br>71.4<br>80.3	72.0 ± 7.9
Medium + MET + Cu 2.5 mM	985<br>962<br>978	975 ± 11	98.5<br>96.2<br>97.8	97.5 ± 1.1
Medium + MET + Cu 2.5 mM<br>LDPE 2.5 g L^{-1}	765<br>912<br>954	877 ± 99	76.5<br>91.2<br>95.4	87.7 ± 9.9
Medium + MET + Cu 2.5 mM<br>LDPE 5 g L^{-1}	784<br>775<br>802	787 ± 13	78.4<br>77.5<br>80.2	78.7 ± 1.3
Medium + MET + Cu 5 mM	848<br>743<br>892	827 ± 76	84.8<br>74.3<br>89.2	82.7 ± 7.6
Medium + MET + Cu 5 mM<br>LDPE 2.5 g L^{-1}	1017<br>775<br>965	919 ± 127	101.7<br>77.5<br>96.5	91.9 ± 12.7
Medium + MET + Cu 5 mM<br>LDPE 5 g L^{-1}	743<br>1019<br>945	902 ± 142	74,3<br>101.9<br>94.5	90.2 ± 14.2
